# Exploring the association between pro-inflammatory diets and chronic liver diseases: evidence from the UK Biobank

**DOI:** 10.3389/fnut.2025.1537855

**Published:** 2025-01-27

**Authors:** Lili Pan, Zhengrong Xu, Yining Li, Guoen Cai, Haibing Gao, Shenglong Lin

**Affiliations:** ^1^Fujian Provincial Key Laboratory on Hematology, Fujian Institute of Hematology, Fujian Medical University Union Hospital, Fuzhou, China; ^2^Translational Medicine Center on Hematology, Fujian Medical University, Fuzhou, China; ^3^Department of Neurology, Fujian Medical University Union Hospital, Fuzhou, China; ^4^Department of Severe Hepatopathy, Mengchao Hepatobiliary Hospital of Fujian Medical University, Fuzhou, China

**Keywords:** dietary inflammatory index, pro-inflammatory diet, dietary pattern, chronic liver diseases (CLD), UK Biobank

## Abstract

**Background:**

Chronic liver diseases (CLD) continue to pose a significant global burden, potentially exacerbated by pro-inflammatory diets. This study explores the relationship between the Dietary Inflammatory Index (DII), a measure of dietary inflammatory potential, and CLD risk.

**Methods:**

Utilizing data from the UK Biobank cohort, we assessed the dietary information and calculated the DII for each participant. Cox proportional hazards models and Fine-Gray competing risk models were employed to evaluate the association between DII and CLD incidence, adjusting for potential confounders.

**Results:**

Our analysis included 121,329 participants with a median follow-up of 604.43 weeks, during which 4,018 developed CLD. A higher DII, indicating a more inflammatory diet, was associated with a 16% increased risk of CLD [hazard ratio (HR) = 1.162, *P* = 0.001], with each unit increase in DII elevating the risk by 3.3% (HR: 1.033, *P* < 0.001). A significant linear association between DII and CLD was observed. Competing risk analyses, which accounted for cirrhosis, liver cancer, and death, supported these findings. Subgroup analyses confirmed the robustness of the DII's association across various demographic and lifestyle factors. Moreover, a higher DII was positively associated with the progression of CLD to cirrhosis. Sensitivity analyses, including energy-adjusted DII and typical dietary DII, reinforced our results. Additionally, adherence to anti-inflammatory dietary patterns, as indicated by higher Healthy Eating Index 2020 and Mediterranean Diet Score values, was inversely associated with CLD risk.

**Conclusion:**

Our study highlights the potential benefits of adopting anti-inflammatory diets as a strategy for the prevention and management of CLD. Comprehensive dietary interventions may play a pivotal role in mitigating the global burden of CLD.

## 1 Introduction

Chronic liver diseases (CLD) remain a significant global health burden, responsible for ~2 million deaths annually, ranking as the 11th leading cause of death and the 15th leading cause of disability-associated life-years worldwide (1). Although recent progress in viral hepatitis prevention and treatment, such as hepatitis B and hepatitis C, the challenge remains substantial, particularly in developing countries (2). This issue is further compounded by the increasing prevalence of non-alcoholic fatty liver disease (NAFLD), now termed metabolic dysfunction-associated fatty liver disease (MAFLD) or metabolic dysfunction-associated steatotic liver disease (MASLD), driven by rising metabolic risk factors such as obesity and diabetes. These conditions are projected to more than double the incidence of advanced liver diseases by 2030, exacerbated by worrying trends in obesity among children and adolescents, which significantly heighten the likelihood of liver disease in later life (3). The economic impact is similarly profound, with liver disease incurring a cost of $32.5 billion in the United States in 2016 alone (1). These significant health and economic challenges necessitate coordinated global efforts to manage the burden of CLD and mitigate its growing impact worldwide.

CLD is intricately linked to the liver's role as the largest internal organ, pivotal in metabolic processes. Responsible for the metabolism of carbohydrates, fats, and proteins, as well as detoxification and hormone production, the liver's function is profoundly affected by diet (4). Diets high in free sugars, saturated fats, and excess calories can exacerbate fat accumulation in the liver, contributing to NAFLD. Intrahepatic triglyceride (IHTG) synthesis primarily relies on fatty acids in the liver, but it can also arise from non-lipid dietary sources, such as excessive free sugar intake. These substrates are converted into saturated fatty acids (SFAs) via hepatic *de novo* lipogenesis (5). A larger cohort study involving Chinese adults (*n* = 4,365) revealed that patients with NAFLD (diagnosed by ultrasonography) consumed a diet richer in carbohydrates and free sugars than participants without NAFLD (6). The consumption of free sugars, particularly fructose, has been linked to liver cancer development in another cohort study (7). Fructose has been implicated in the development of liver cancer, with high fructose intake shown to promote hepatocellular carcinoma (HCC) through the enhancement of O-GlcNAcylation mediated by microbiota-derived acetate in an HCC mouse model (8). Additionally, in a diethylnitrosamine (DEN)-induced liver tumor model, dietary fructose enhanced the proliferation, invasion, and tumorigenicity of hepatic progenitor cells, providing further mechanistic support for fructose's role in liver tumorigenesis (9). However, it is noted that some studies have yielded inconsistent results (10, 11). This inconsistency may be due to the diverse and complex dietary habits influenced by geographic, ethnic, and cultural differences.

Investigating overall dietary patterns, rather than individual foods, may provide deeper insights into the relationship between nutrition and the risk of CLD. The prevalent “Western dietary pattern” in modern society, characterized by desserts and processed meats, is a contributing factor to the rising incidence of CLD in recent years (1, 12). Pro-inflammatory diets, measured using the Dietary Inflammatory Index (DII) (13), have been linked to several chronic diseases (14–16). While several studies have assessed the association between DII and CLD risk, most have been limited by a focus on specific diseases, small sample sizes or cross-sectional designs (17–21). A recent study using data from the UK Biobank indicated that diets with high energy-adjusted DII (eDII) increased the risk of severe NAFLD [hazard ratio (HR): 1.19; 95% CI: 1.03–1.38] (22). On the other hand, CLD includes diverse conditions such as alcoholic liver disease, drug-induced liver injury (DILI), autoimmune liver disease, cirrhosis, and liver cancer. Given that inflammation is a shared pathological feature in CLD, the DII score may influence a broader range of liver conditions. However, the link between DII and the full spectrum of CLD remains unclear, highlighting the need for more comprehensive research to address this gap.

Therefore, we aim to investigate the relationship between the DII and the incidence of all types of CLD in a large, prospective, long-term follow-up cohort of UK Biobank, seeking to provide a more comprehensive understanding of how pro-inflammatory diets may influence overall liver health.

## 2 Methods

### 2.1 Study design and participants

This prospective cohort study used data from the UK Biobank, a large population-based study of ~500,000 individuals aged 37–73 years, recruited between 2006 and 2010 from 22 assessment centers across England, Scotland, and Wales (23). Participants provided informed consent, and ethical approval was granted by the North West Multi-Center Research Ethics Committee. At baseline, all participants visited an assessment center, where they provided information about their medical history and lifestyle, underwent a physical examination, and submitted urine and blood samples. Further details regarding the UK Biobank protocol are available online at http://www.ukbiobank.ac.uk.

### 2.2 DII calculation

Dietary intake was assessed using the Oxford WebQ, an online 24-h dietary recall questionnaire, which was administered to participants at different time points between February 2011 and April 2012. The Oxford WebQ captures information on the consumption of over 200 common food items and beverages, automatically generating estimates of energy and nutrient intake (24). The average nutrient intake across different time points was used in this study to calculate participants' DII. The validity of this questionnaire for nutritional assessment has been demonstrated in multiple prior clinical studies (19, 25). The DII is a scoring system developed to assess the inflammatory potential of an individual's diet based on a variety of food and nutrient components. In this study, the DII was calculated using 29 dietary parameters available from the UK Biobank dataset (Supplementary Table S1). Each component was weighted based on its known pro-inflammatory or anti-inflammatory effects, with reference to a global mean and standard deviation (SD) obtained from the literature. For each dietary component, a *Z-*score was calculated by comparing an individual's intake to the global mean. Specifically, the global mean intake was subtracted from the individual's average intake, and the difference was divided by the global SD. This *Z-*score reflects how much the individual's intake deviates from the global average, standardized by the variation within the global population. The *Z-*scores were then converted to percentiles, ranging from −1 to 1, to ensure comparability with the global reference data. Next, each percentile score was multiplied by the component's inflammatory weight (based on its overall inflammatory effect) to calculate the score for each component. Finally, the DII for each individual was computed as the sum of the component-specific DII scores. Additionally, the eDII was calculated by normalizing nutrient intake using the density method (nutrient intake per 1,000 kcal of total energy). The remaining calculation steps followed the same process as the DII calculation (25).

### 2.3 Inclusion and exclusion criteria

In this research, an average of five 24-h dietary recalls was utilized, with data collected between April 2009 (the 1st instance) and June 2012 (the 4th instance), as detailed on the UK Biobank website (UKB category 100090). Participants from the UK Biobank who completed at least one online 24-h dietary recall questionnaire were included, with the start date determined by the completion date of the first questionnaire. The exclusion criteria were as follows: (1) participants who were missing vital nutrient component data, characteristic data, or personal lifestyle data; (2) participants with a history of CLD or malignant tumors, defined by self-reported medical conditions (UKB field IDs 20001 and 20002), and those diagnosed with CLD or a malignant tumor prior to the baseline; and (3) given the chronic nature of CLD, a 1-year landmark analysis was used to exclude participants who experienced relevant events within the first year of the study.

### 2.4 Outcome determination

The primary outcome of this study was the incidence of CLD, which includes conditions such as fatty liver disease, hepatitis, cirrhosis, liver fibrosis, and HCC. CLD cases were identified through linked hospital records (UKB category 2002) and cancer registries (UKB category 100092) data, using International Classification of Diseases (ICD-10) codes listed in the Supplementary Table S2. Incident cases were defined as those with a first diagnosis of CLD (UKB category 1712) during follow-up, and deaths of participants were captured through national death registries (UKB category 100093).

### 2.5 Baseline covariates and reclassification

Baseline covariates were collected via self-reported questionnaires and physical measurements from the UK Biobank database. These included age, sex, ethnicity, education, smoking and alcohol consumption status, physical activity levels, body mass index (BMI), blood pressure, and diabetes status. Socioeconomic status was measured using the Townsend deprivation index (TDI), which is derived from participants' residential postal codes and reflects local unemployment, home ownership, and overcrowding rates. Age was classified into younger or older group by the median age (58 years old); TDI was also divided into high or low by the median level (−2.3); Participants' educational qualifications (UKB field ID 6138) were reclassified into three broader categories: High, Median, and Low. The High Education category included participants who reported having a College or University degree (original code: 1). The Median Education category included those with intermediate qualifications such as Advanced Level/Advanced Subsidiary Level or equivalent (code 2), National Vocational Qualification, Higher National Diploma, or Higher National Certificate or equivalent (code 5), and Other professional qualifications such as nursing or teaching (code 6). The Low Education category comprised participants with Ordinary Level/General Certificate of Secondary Education or equivalent (code 3), Certificate of Secondary Education or equivalent (code 4), and those reporting None of the above (code 7), as well as individuals who selected Prefer not to answer (code 3); participants' blood pressure levels were reclassified into 4 categories based on systolic pressure (SP) and diastolic pressure (DP), following standard clinical guidelines. The categories were: Normal, Elevated, Stage 1 Hypertension, and Stage 2 Hypertension. Participants with an SP of <120 mmHg and a DP of <80 mmHg were classified as “Normal”. Those with an SP between 120 and 129 mmHg and a DP of <80 mmHg were classified as “Elevated”. Individuals were classified as “Stage 1 Hypertension” if they had an SP between 130 and 139 mmHg or a DP between 80 and 89 mmHg. Finally, those with an SP of 140 mmHg or higher, or a DP of 90 mmHg or higher, were classified as “Stage 2 Hypertension”.

### 2.6 Statistical analyses

In the baseline characteristic comparison, categorical variables were presented as frequencies and proportions, while continuous variables were depicted as means with SDs or medians with interquartile ranges (IQR). The analysis of categorical variables employed the Pearson chi-square test, whereas continuous variables were compared using the Analysis of Variance (ANOVA) for normally distributed variables or the Kruskal-Wallis test for non-normally distributed data. Cox proportional hazards (PH) regression models were used to examine the association between DII and the risk of developing CLD. HRs and 95% confidence intervals (CIs) were calculated to estimate the risk of CLD across quartiles of DII. The models were adjusted for potential confounders, including age, sex, ethnicity, education, physical activity, BMI, smoking status, alcohol consumption, diabetes, and socioeconomic status. To evaluate a linear trend, the median of each quartile of the DII was treated as a continuous variable in each model. Additionally, restricted cubic splines were applied to assess potential non-linear associations between DII and CLD risk. The proportional hazards assumption was tested using Schoenfeld residuals. The PH assumption test was used to access the Cox PH models confirming that HRs of each covariate should remain constant over time. For those models failed to pass the PH assumption test, the alternative accelerated failure time (AFT) models, which do not rely on the PH assumption test (26), were applied. Considering that the occurrences of cirrhosis, liver cancer and death were the competing events for CLD, the Fine-Gray models were used to calculate subdistribution HR (sHR) for further depicting the relationship between DII and CLD risk. To explore disease progression, participants who developed CLD were analyzed for cirrhosis or liver cancer risk, with the follow-up period defined as the time from CLD diagnosis to cirrhosis or liver cancer occurrence.

To validate the relationship between DII and CLD risk, subgroup analyses were performed for each category of covariables. Sensitivity analyses were also conducted, including the similar analysis with eDII, the association of DII and inflammation indexes, and the participant with typical dietary pattern (UKB field ID 100020) to reduce the potential changes in dietary patterns over time. Furthermore, two dietary pattern scoring system, including the healthy eating index 2020 (HEI-2020) (27) and the Mediterranean diet score (MEDS) (28), were calculated to investigate the link between pro-inflammation dietary and CLD (Supplementary Tables S3, S4). For all the above analyses, a *P*-value of < 0.05 was considered statistically significant. All analyses in this study, including data management, statistical analysis, model construction, and graph plotting, were conducted using R statistical software (version 4.3.1; R Foundation Inc.; http://cran.r-project.org/).

## 3 Results

### 3.1 Characteristics of participants

**Figure 1:**
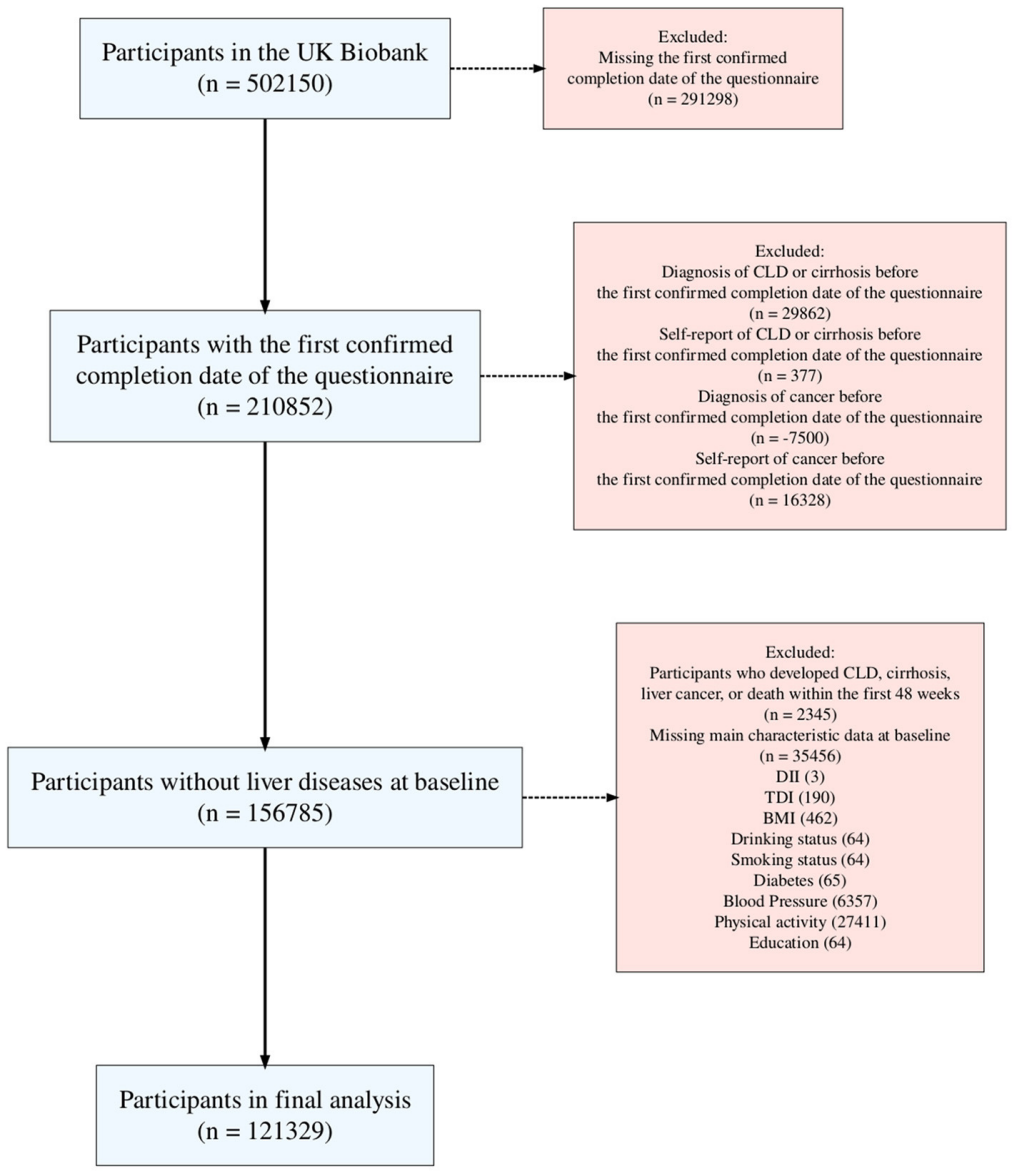
The flowchart of participant selection.

A total of 121,329 participants were included in this study (Figure 1), with a median follow-up time of 604.43 (IQR 569.14–646) weeks. During the follow-up period, 4,018 participants developed CLD, including 1,168 (29.07%) with NAFLD, 131 with biliary liver disease, 172 with alcoholic disease, 73 with viral liver disease, 60 with autoimmune liver disease, and 19 with DILI. Liver disease types with small case numbers, rare or less common liver diseases, and those that could not be classified under specific etiologies were grouped into the “Others” category. Table 1 presents the baseline characteristics of the participants in this study according to the quartiles of DII. Participants in the high DII group were more likely to be younger, female, and from areas with higher deprivation. They tended to have higher BMI, lower education levels, and were more likely to be current drinkers and smokers and less physically active. While differences in diabetes prevalence and blood pressure categories were statistically significant, the absolute differences were small.

**Table 1:** Characteristics of Participants according to the quartiles of DII.

	**Total (*n* = 121,329)**	**Q1 (*n* = 30,333)**	**Q2 (*n* = 30,332)**	**Q3 (*n* = 30,332)**	**Q4 (*n* = 30,332)**	***P***
**CLD**						
Cases/participants	4,018/117,311	962/29,371	920/29,412	986/29,346	1,150/29,182	
**Cirrhosis**						
Cases/participants	296/121,033	62/30,271	55/30,277	82/30,250	97/30,235	
**Liver cancer**						
Cases/participants	185/121,144	54/30,279	35/30,297	43/30,289	53/30,279	
Follow-up (weeks)	604.43 (569.14, 646)	604.14 (568.29, 646.29)	604.29 (569.71, 645)	604.43 (569.86, 645)	604.57 (569.29, 647.57)	0.005
Age, *n* (%)						< 0.001
Older	57,821 (48)	16,227 (53)	15,019 (50)	14,112 (47)	12,463 (41)	
Younger	63,508 (52)	14,106 (47)	15,313 (50)	16,220 (53)	17,869 (59)	
Sex, *n* (%)						< 0.001
Female	63,575 (52)	14,828 (49)	15,463 (51)	16,066 (53)	17,218 (57)	
Male	57,754 (48)	15,505 (51)	14,869 (49)	14,266 (47)	13,114 (43)	
TDI, *n* (%)						< 0.001
High	60,380 (50)	14,722 (49)	14,569 (48)	14,919 (49)	16,170 (53)	
Low	60,949 (50)	15,611 (51)	15,763 (52)	15,413 (51)	14,162 (47)	
Ethnicity, *n* (%)						< 0.001
White	115,596 (95)	29,026 (96)	29,276 (97)	29,065 (96)	28,229 (93)	
Others	5,733 (5)	1,307 (4)	1,056 (3)	1,267 (4)	2,103 (7)	
BMI	26.3 (23.81, 29.34)	26.03 (23.59, 28.98)	26.1 (23.7, 29.03)	26.35 (23.9, 29.34)	26.76 (24.1, 29.97)	< 0.001
Alcohol, *n* (%)						< 0.001
Rare	3,797 (3)	858 (3)	774 (3)	873 (3)	1,292 (4)	
Previous	3,591 (3)	827 (3)	754 (2)	852 (3)	1,158 (4)	
Current	113,941 (94)	28,648 (94)	28,804 (95)	28,607 (94)	27,882 (92)	
Smoking, *n* (%)						< 0.001
Rare	68,079 (56)	17,091 (56)	17,339 (57)	17,025 (56)	16,624 (55)	
Previous	43,751 (36)	11,395 (38)	11,019 (36)	10,955 (36)	10,382 (34)	
Current	9,499 (8)	1,847 (6)	1,974 (7)	2,352 (8)	3,326 (11)	
PA, *n* (%)						< 0.001
No reach	21,702 (18)	4,088 (13)	5,072 (17)	5,911 (19)	6,631 (22)	
Reach	99,627 (82)	26,245 (87)	25,260 (83)	24,421 (81)	23,701 (78)	
Education, *n* (%)						< 0.001
Low	27,360 (23)	6,214 (20)	6,018 (20)	6,797 (22)	8,331 (27)	
Median	40,983 (34)	9,975 (33)	9,958 (33)	10,413 (34)	10,637 (35)	
High	52,986 (44)	14,144 (47)	14,356 (47)	13,122 (43)	11,364 (37)	
BP, *n* (%)						< 0.001
Normal	17,908 (15)	4,132 (14)	4,306 (14)	4,481 (15)	4,989 (16)	
Elevated	22,497 (19)	5,302 (17)	5,655 (19)	5,682 (19)	5,858 (19)	
Stage 1	45,474 (37)	11,714 (39)	11,534 (38)	11,274 (37)	10,952 (36)	
Stage 2	35,450 (29)	9,185 (30)	8,837 (29)	8,895 (29)	8,533 (28)	
Diabetes, *n* (%)						< 0.001
No	116,189 (96)	29,038 (96)	29,121 (96)	29,107 (96)	28,923 (95)	
Yes	5,140 (4)	1,295 (4)	1,211 (4)	1,225 (4)	1,409 (5)	

TDI, townsend deprivation index; PA, physical activity.

### 3.2 DII and CLD risk

**Figure 2:**
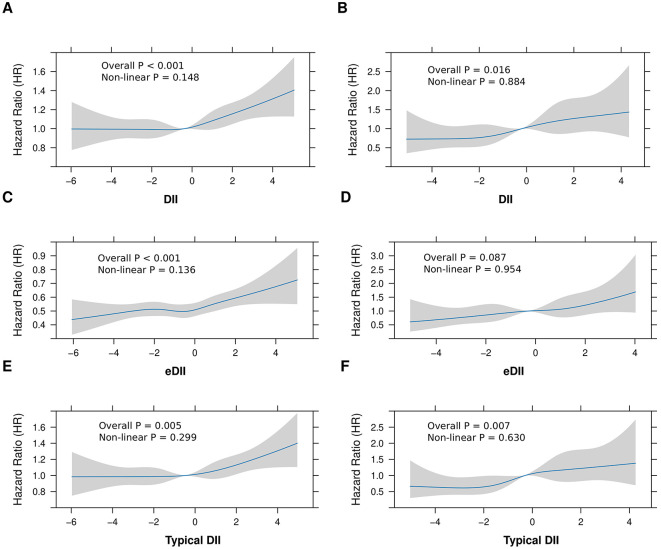
Non-linear associations between dietary inflammatory indices and risk of CLD and Cirrhosis. This figure illustrates the associations between dietary inflammatory indices (DII, eDII, and DII from typical dietary) and hazard ratios (HR) for CLD and cirrhosis. **(A, B)** Depict the relationships between DII and the risk of CLD **(A)** and cirrhosis **(B)**. Similarly, **(C, D)** present the associations for eDII with CLD and cirrhosis, respectively, while **(E, F)** show the effects of DII from typical dietary on the risks of CLD **(E)** and cirrhosis **(F)**. The *blue lines* represent the estimated HRs, with shaded regions denoting 95% confidence intervals. Statistical significance and deviations from linearity are evaluated using both overall and non-linear *P*-values.

The analysis of the association between DII and CLD incidence (Table 2) revealed a significant correlation. Individuals with higher DII levels (Q4 vs. Q1) exhibited an increased risk of developing CLD (HR: 1.162; 95%CI: 1.065–1.268; *P* = 0.001) based on the Cox PH model (Model 3), after adjusting for various confounding factors such as age, sex, race, educational level, TDI, alcohol use, smoking, BMI, physical activity, blood pressure, and diabetes. When DII was treated as a continuous variable, the HR for CLD risk was 1.033 (95% CI: 1.017–1.050; *P* < 0.001), adjusted for the same factors. Furthermore, trend analysis indicated a significant positive linear association between DII and CLD risk (*P* for trend < 0.001). Conversely, no statistically significant non-linear relationship was detected (*P* = 0.148), suggesting that the association between DII and CLD is predominantly linear (Figure 2A). To further explore this association, competing risk analyses were applied, considering cirrhosis, liver cancer and death as competing events. After adjustment for the aforementioned covariates (Model 3), the results were consistent with the Cox PH model: participants in the highest DII quartile exhibited an 14.5% higher risk of CLD compared to those in the lowest quartile (sHR = 1.145, 95% CI: 1.049–1.249, *P* = 0.002). Additionally, each unit increase in DII was associated with a 3.0% rise in CLD risk (sHR = 1.030, 95% CI: 1.013–1.047, *P* = 0.001).

**Table 2:** Associations of DII and CLD risk.

**Models**	**Q1**	**Q2**	**Q3**	**Q4**	**Continous**	***P* for trend**
**Cox regression**						
Model 1	1 (reference)	0.98 (0.895–1.073) 0.66	1.062 (0.971–1.161) 0.186	1.26 (1.156–1.374) < 0.001	1.051 (1.034–1.068) < 0.001	< 0.001
Model 2	1 (reference)	0.97 (0.886–1.062) 0.511	1.025 (0.938–1.121) 0.58	1.16 (1.064–1.266) 0.001	1.033 (1.016–1.050) < 0.001	< 0.001
Model 3	1 (reference)	0.971 (0.887–1.062) 0.517	1.027 (0.94–1.123) 0.554	1.162 (1.065–1.268) 0.001	1.033 (1.017–1.050) < 0.001	< 0.001
**Fine-gray**						
Model 1	1 (reference)	0.976 (0.892–1.069) 0.6	1.049 (0.96–1.147) 0.29	1.229 (1.127–1.34) < 0.001	1.045 (1.028–1.063) < 0.001	/
Model 2	1 (reference)	0.968 (0.885–1.06) 0.49	1.017 (0.93–1.112) 0.71	1.139 (1.044–1.242) 0.004	1.028 (1.012–1.046) 0.001	/
Model 3	1 (reference)	0.97 (0.886–1.062) 0.51	1.02 (0.933–1.115) 0.66	1.145 (1.049–1.249) 0.002	1.030 (1.013–1.047) 0.001	/

Model 1, Adjusted for age, sex, ethnicity, education, and the Townsend Deprivation Index; Model 2, Further adjusted for drinking status, smoking status, body mass index, and physical activity in addition to the variables in Model 1; Model 3, Includes adjustments for all variables in Model 2, with additional consideration for diabetes and blood pressure status; Cox regression Model: the hazard ratios (HR) are reported; Fine-Gray Models: Adjustments are made for cirrhosis, liver cancer, and death, with subdistribution hazard ratios (sHR) reported; Data are presented as HR or sHR with 95% confidence intervals and the corresponding p-values.

**Figure 3:**
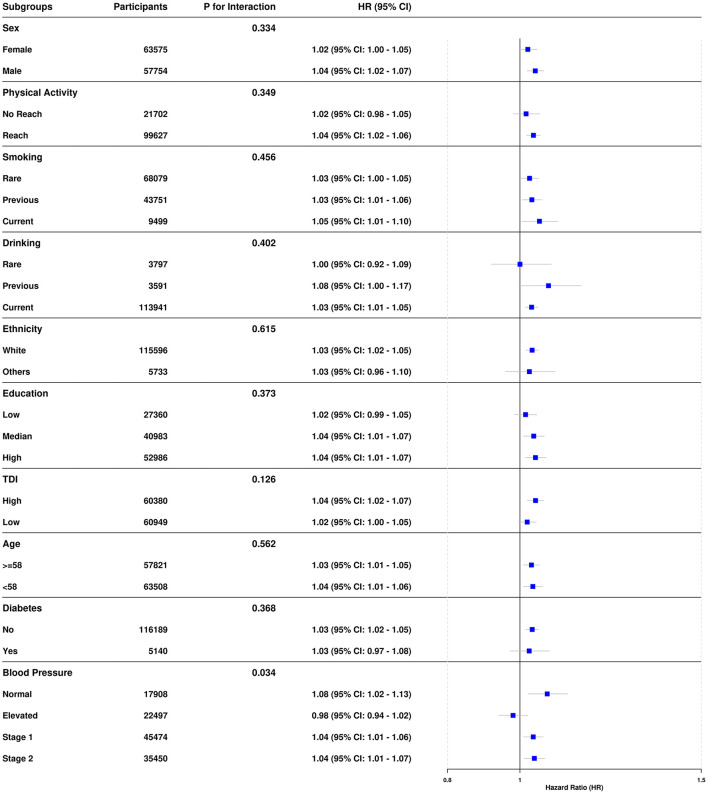
Associations between DII and CLD risk across subgroups. Hazard ratios (HR) and 95% confidence intervals for various subgroups, adjusted for potential confounders including age, sex, ethnicity, education level, Townsend deprivation index, smoking, drinking, physical activity, diabetes status, and blood pressure categories. Interaction *P*-values assess heterogeneity across subgroups. A reference HR of 1.0 indicates no effect, with deviations suggesting increased or decreased risk within each subgroup. Subgroup comparisons are visualized using forest plots for clarity.

In the subgroup analyses (Figure 3), the association between the DII score and the risk of CLD generally remained consistent across various categories, including sex (*P* for interactio*n* = 0.334), physical activity (*P* for interactio*n* = 0.349), smoking status (*P* for interactio*n* = 0.456), drinking status (*P* for interactio*n* = 0.402), ethnicity (*P* for interactio*n* = 0.615), education level (*P* for interactio*n* = 0.373), TDI (*P* for interactio*n* = 0.126), age (*P* for interactio*n* = 0.562), and diabetes status (*P* for interactio*n* = 0.368), indicating no significant differences in the DII and CLD association within these subgroups. However, a significant interaction was observed with blood pressure (*P* for interactio*n* = 0.034), where higher HR among those with Stage 1 and Stage 2 blood pressure suggest that the risk of CLD associated with higher DII scores may be more pronounced in individuals with elevated blood pressure levels, indicating a potential moderating effect of hypertension on this relationship.

To examine the influence of the DII on the progression of CLD to cirrhosis or liver cancer, a separate analysis was conducted on those 4,018 CLD patients. The analysis indicated that the DII score, assessed both as quartiles and a continuous variable, significantly correlate with the risk of cirrhosis development (highest vs. lowest quartile: HR = 1.583, 95% CI: 1.139–2.199, *P* = 0.006; continuous DII: HR = 1.110, 95% CI: 1.045–1.178, *P* < 0.001). For liver cancer, no significant association was found between DII and progression (Table 3; Figure 2B). These findings suggest that while DII may contribute to CLD and cirrhosis development, it does not appear to significantly influence progression to liver cancer in this cohort.

**Table 3:** Associations between dietary inflammatory indices and dietary patterns with risk of cirrhosis and liver cancer.

**Models**	**Q1**	**Q2**	**Q3**	**Q4**	**Continous**	***P* for trend**
**DII**						
Cirrhosis	1 (reference)	1.074 (0.743–1.552) 0.705	1.374 (0.980–1.927) 0.065	1.583 (1.139–2.199) 0.006	1.110 (1.045–1.178) < 0.001	0.001
^*^Liver cancer	1 (reference)	0.551 (0.267–1.137) 0.069	0.714 (0.387–1.318) 0.275	0.905 (0.497–1.648) 0.735	0.997 (0.885–1.123) 0.954	0.513
**eDII**						
Cirrhosis	1 (reference)	1.190 (0.825–1.716) 0.352	1.176 (0.828–1.671) 0.365	1.486 (1.062–2.079) 0.021	1.010 (1.028–1.176) 0.006	0.017
^*^Liver cancer	1 (reference)	1.123 (0.599–2.107) 0.716	1.167 (0.633–2.153) 0.624	0.878 (0.448–1.721) 0.690	0.974 (0.860–1.102) 0.682	0.402
**DII (typical dietary)**						
Cirrhosis	1 (reference)	1.178 (0.753–1.841) 0.474	1.846 (1.248–2.730) 0.002	1.902 (1.291–2.801) 0.001	1.134 (1.059–1.214) < 0.001	0.001
^*^Liver cancer	1 (reference)	0.600 (0.370–0.973) 0.038	0.870 (0.568–1.334) 0.524	0.840 (0.545–1.294) 0.430	1.003 (0.924–1.090) 0.936	0.78
**HEI2020**						
Cirrhosis	1 (reference)	0.749 (0.544–1.032) 0.077	0.939 (0.689–1.280) 0.689	0.874 (0.631–1.211) 0.419	0.994 (0.982–1.006) 0.317	0.524
^*^Liver cancer	1 (reference)	0.647 (0.429–0.978) 0.039	0.970 (0.662–1.421) 0.875	0.720 (0.471–1.103) 0.131	0.993 (0.977–1.008) 0.346	0.3
**MEDS**						
Cirrhosis	/	/	/	/	0.896 (0.827–0.971) 0.007	/
^*^Liver cancer	/	/	/	/	1.007 (0.856–1.153) 0.931	/

^*^Accelerated Failure Time model; DII, dietary inflammatort index; eDII; energy-adjuested DII; HEI2020, Healthy Eating Index 2020; MEDS, Mediterranean Diet Score; All models were adjusted for age, sex, ethnicity, education, the Townsend Deprivation Index, drinking status, smoking status, body mass index, physical activity, diabetes and blood pressure status; The Cox regression model reports hazard ratios (HRs), indicating effects on hazard; The AFT models provide adjusted time ratios from model coefficients, reflecting time scaling; Data include 95% confidence intervals and *P*-values.

To further validate the association between the DII and CLD risk, several sensitivity analyses were conducted. The eDII showed similar associations with increased CLD risk (highest vs. lowest quartile: HR = 1.138, 95% CI: 1.042–1.244, *P* = 0.004; continuous eDII: HR = 1.036, 95% CI: 1.017–1.054, *P* < 0.001), supporting the robustness of primary findings (Supplementary Table S5, Model 3; Figure 2C). Additionally, analysis of a subset of participants reporting typical dietary intake revealed consistent results, demonstrating higher DII scores associated with increased CLD risk (Supplementary Table S5, Model 3; Figure 2E). Both eDII and DII from typical dietary presented a significant positive relationship with cirrhosis progression, but not with liver cancer in CLD (Table 3; Figures 2D, F). Significant yet weak positive correlations were observed between the DII and inflammatory biomarkers, including white blood cells (WBC: *r* = 0.062), neutrophils (NE: *r* = 0.054), and C-reactive protein (CRP: *r* = 0.053), with all *P*-values < 0.05, suggesting that the DII effectively measures dietary inflammation. These sensitivity analyses collectively reinforce the link between higher dietary inflammatory potential and an increased CLD risk across different analytical approaches and subgroups.

### 3.3 Dietary pattern and CLD risk

After adjusting for multiple covariates, including age, sex, ethnicity, education, TDI, alcohol consumption, smoking history, BMI, physical activity, diabetes status, and blood pressure, both the HEI-2020 and the MEDS remained significantly associated with CLD risk. Specifically, individuals in the highest quartile of HEI-2020 had a significantly reduced risk of CLD compared to those in the lowest quartile (HR = 0.923, 95% CI: 0.860–0.992, *P* = 0.016), and each unit increase in the continuous HEI-2020 score was associated with a modest yet significant decrease in CLD risk (HR = 0.995, 95% CI: 0.992–0.998, *P* = 0.002) (Supplementary Table S6; Supplementary Figure 1A). Similarly, higher adherence to the Mediterranean diet, as measured by the MEDS, was linked to an reduced risk of CLD (HR = 0.961. 95% CI: 0.992–0.998, *P* < 0.001) (Supplementary Table S7; Supplementary Figure 1C). These results are consistent with the findings from the DII, highlighting the importance of overall dietary patterns in influencing CLD risk. Notably, only MEDS showed a significant negative link to cirrhosis development (Supplementary Figures 1B, D). Neither the HEI-2020 nor MEDS were significantly related to liver cancer (Table 3).

## 4 Discussion

This study investigated the association between the DII and the risk of CLD in a large prospective cohort of 121,329 participants, followed for over 12 years. The primary findings demonstrated a significant positive correlation between higher DII scores and an elevated risk of CLD, with individuals in the highest DII quartile exhibiting a 16.2% greater risk compared to those in the lowest quartile. This relationship persisted across sociodemographic, lifestyle, and health-related subgroups. Competing risk analyses, which accounted for outcomes such as cirrhosis, liver cancer, and death, produced consistent results, reinforcing the sustained positive correlation between higher dietary inflammatory potential (as indicated by elevated DII scores) and increased CLD risk. Moreover, DII was positively associated with the progression of CLD to cirrhosis, but not to liver cancer. Sensitivity analyses, including models adjusted for total energy intake and typical dietary, further validated these findings. Overall, the study suggests that a more pro-inflammatory diet is associated with an increased risk of developing CLD, underscoring the potential role of dietary inflammation in liver health.

There is a close connection between the gut and the liver. The enterohepatic tissues are organized into multiple layers of physical, chemical, microbial, and immunological barriers that play a crucial role in maintaining intestinal homeostasis. These barriers serve to regulate the movement of intestinal antigens, microbial components, and microorganisms, thereby preventing their translocation and limiting their spread to other organs, particularly the liver. The concept of a Gut–Liver axis was put forward to emphasize the clinically relevant link between gut and liver diseases, initially to describe antibodies directed against intestinal microorganisms and food antigens in the circulation of patients with CLD (4, 29, 30). Once materials (including microorganisms and food antigens) cross the gut epithelium, various immune cells, including innate lymphoid cells, invariant T cells, and T cell subsets, interact with gut luminal contents and microbiota, helping regulate gut homeostasis and protective immune responses. Mononuclear phagocytes (e.g., macrophages and dendritic cells) play a crucial role in directly handling foreign material and producing antibodies, notably IgA, that affect gut antigen uptake and response (31, 32). In addition to the immune control conducted by phagocytes and antibodies, materials that evade direct immune regulation must still traverse the vascular endothelium before entering the circulatory system. The gut-vascular barrier (GVB) is crucial for preventing bacterial translocation from the intestine to the liver (33, 34). Disruption of this barrier has been linked to the pathogenesis of NAFLD (35), and liver metastasis in colorectal cancer (36). Certain gut luminal contents, such as live commensal and pathogenic microorganisms, as well as hormones, cytokines, bacterial Pathogen-associated molecular patterns (PAMPs), and metabolites, can cross the gut barrier and enter the bloodstream, where they are transported to the liver via portal blood (37). The hepatic immune system, including Kupffer cells and dendritic cells, works similarly to its gut counterpart by trapping and processing antigens, thus preventing their spread throughout the body (38). Impairment of intestinal barrier could lead to progression of CLD by increasing hepatic inflammation, fibrosis, and portal hypertension, meanwhile further weakens intestinal barrier integrity and exacerbates the gut-liver axis dysregulation. In advanced stages of CLD, the rise in portal pressure and gut-derived systemic inflammation increases the risk of multiple organ failure, worsening complications and mortality (39).

The stability of the gut microbiota is critical to maintaining intestinal barrier function and preventing liver disease progression, including HCC, DILI, and viral hepatitis. In hepatocarcinogenesis, disrupted gut microbiota and translocated lipopolysaccharides (LPS) promote cancer development through the Toll-like receptor (TLR4)-dependent pathways (40). Genetically driven dysbiosis, such as a deficiency in NACHT, LRR, and PYD domains protein 6 (NLRP6), exacerbates steatohepatitis (41), while obesity-induced dysbiosis promotes HCC formation through the cytotoxic effects of secondary bile acids (42, 43). Gut-derived bile acids influence hepatic immune surveillance by recruiting natural killer T cells (44, 45). In NAFLD-related HCC, dysbiosis is linked to systemic inflammation, with fecal microbiota from these patients suppressing T cell responses, and microbial DNA in cirrhotic livers correlating with immune exhaustion (42). In DILI, interventions targeting gut dysbiosis, such as LPS-binding peptides or probiotics, have shown efficacy in ameliorating conditions like acetaminophen-induced injury (46). Long-term use of antibiotics or proton pump inhibitors, indicative of gut dysbiosis, is associated with a higher risk of acute liver failure (47). In the context of viral hepatitis, the gut microbiota plays a critical role in facilitating hepatitis B virus clearance via TLR4 signaling pathways (48). Additionally, in hepatitis C virus-related cirrhosis, disruption in gut fatty acid metabolism was observed (49). Generally, dietary patterns could influence gut microbial stability, highlighting the importance of nutrition in managing liver diseases.

Common dietary pattern assessments include the DII, HEI-2020, and MEDS (50). The DII specifically measures the inflammatory potential of the diet, which evaluates how food components and nutrients either promote or alleviate inflammation. In our study, a higher DII, reflecting a more pro-inflammatory diet, was linked with significantly increased CLD risk. This aligns with the growing evidence around chronic inflammation being a key driver in liver disease progression, including NAFLD and cirrhosis (51, 52). Diets rich in pro-inflammatory components, such as processed foods, refined carbohydrates, and unhealthy fats, may exacerbate liver damage over time through inflammatory pathways (5). In contrast, the HEI-2020 was developed to capture adherence to overall dietary quality as recommended by the Dietary Guidelines for Americans. A higher HEI-2020 score reflects a diet rich in fruits, vegetables, whole grains, lean proteins, and low in added sugars, sodium, and saturated fats (27). Importantly, our results showed that participants in the highest quartile of HEI-2020 scores had significantly lower risks of CLD compared to those in the lowest quartile, and each unit increase in HEI-2020 as a continuous variable was similarly associated with a lower risk of CLD. These findings suggest that overall diet quality, characterized by nutrient-dense and anti-inflammatory foods, offers protection against the development of liver diseases. The MEDS, which measures adherence to the Mediterranean diet, a diet high in plant foods, healthy fats like olive oil, moderate to low in animal products, and low in saturated fats, was also significantly associated with a lower CLD risk. Our results showed that each unit increase in the MEDS corresponded to a reduction in CLD risk. The Mediterranean diet is known for its anti-inflammatory and antioxidant-rich properties, which may delay or prevent liver damage. Our results align with the previous studies in steatotic liver disease and cirrhosis (53, 54). In addition, Guo et al. utilized food frequency questionnaire (FFQ) data from UKB and applied principal component analysis (PCA) to study the effect of dietary patterns on NAFLD, cirrhosis, and liver cancer, showing that the participants with high tertile of Western dietary pattern score had and higher risk of NAFLD, cirrhosis and liver cancer compared with those with low tertile, with increased risk 18%, 21%, and 24%, respectively (12). However, DII is widely validated by researchers, which is considered a relatively reliable and universal tool to assess dietary patterns. A recent meta-analysis, including 10 studies with 242,006 participants from the U.S., UK, Portugal, and Iran, indicated that individuals with higher DII had a significantly increased risk of fatty liver disease (OR 1.63; 95% CI 1.08–2.45) and liver fibrosis (OR 1.15; 95% CI 1.09–1.21) compared to those with lower DII (51). The current study, however, used dietary data from the Oxford WebQ to calculate DII and evaluate its association with CLD risk. Unlike FFQ data, which is designed to capture habitual dietary intake over a long-term period (55), the Oxford WebQ assesses dietary intake over the previous 24 h, allowing for a more accurate estimation of daily food consumption.

Our findings suggest that a pro-inflammatory dietary pattern is associated with a higher risk of CLD, providing further insight into diet's role in liver disease prevention. However, our study did not identify a significant association between the DII and liver cancer, which diverges from the results of a previous prospective multi-center study conducted in the United States, involving 582 participants over a 4-year follow-up period (56). This discrepancy may be attributed to the heterogeneity in tumor development mechanisms, including environmental and genetic factors. Furthermore, variations in participant numbers and follow-up duration could also account for the differences observed between the previous study and our research. Previous study investigating the relationship between diet and liver cancer has also reported similarly null association (10). However, several limitations of this study should be noted: (1) CLD diagnoses were primarily based on participants' hospitalization records, potentially underrepresenting mild or asymptomatic cases that did not seek medical care. (2) The relatively small number of CLD cases may limit the statistical power of the survival models, warranting a cautious interpretation of the results. (3) Baseline exclusion of CLD was partially based on self-reported data, possibly introducing selection bias, although this was mitigated by employing a 1-year landmark analysis. (4) DILI is one of the most common forms of CLD. Due to the wide variety of hepatotoxic substances or drugs linked to DILI, as well as significant inter-individual variability in susceptibility, this study did not include hepatotoxic agents or medications as covariates in the analysis of DII. This could influence the reliability of the results. (5) DII calculations were based on 29 food/nutrient components available in the UK Biobank database, fewer than the 45 components recommended for the original DII assessment, but previous studies have demonstrated that 29–30 components are sufficient to assess dietary inflammatory potential. (6) The UK Biobank participants are predominantly British European and in middle age, possibly limiting the generalizability of the findings to more diverse populations. Therefore, prospective multi-centers studies should be conducted in different countries and ethnic groups in the future.

## 5 Conclusion

In conclusion, our study utilizing data from a large prospective cohort demonstrated that participants following a pro-inflammatory dietary pattern had a significantly higher risk of CLD and an elevated risk of cirrhosis progression among those with CLD. These findings suggest the potential benefits of adhering to an anti-inflammatory diet, which may play a crucial role in both the prevention and management of CLD.

## Data Availability

The data used in this study were obtained from the UK Biobank and are subject to licensing restrictions, making them unavailable for public access. These data were utilized under license specifically for this study. However, they can be provided by the authors upon reasonable request and with the approval of the UK Biobank. Similarly, the R code employed in this study is available from the corresponding authors upon reasonable request.
