# Supplementary material for: Exploring the association between pro-inflammatory diets and chronic liver diseases: evidence from the UK Biobank
Source: Front Nutr. 2025 Jan 27;12:1537855. doi: 10.3389/fnut.2025.1537855 (PMC11807818; doi:10.3389/fnut.2025.1537855)

## *Supplementary Material*

#### 1 Supplementary Tables

**Table S1. Food Parameters and Inflammatory Effect Scores for Dietary Inflammatory Index Calculation in This Study.**

| **Food Parameter** | **Overall Inflammatory Effect Score*** | **Global daily mean intake** | **SD** | **Category** |
| --- | --- | --- | --- | --- |
| Energy (kcal/day) | 0.18 | 2056 | 338 | Macronutrients |
| Carbohydrate (g/day) | 0.097 | 272.2 | 40 |  |
| Total Protein (g/day) | 0.021 | 79.4 | 13.9 |  |
| Total Fat (g/day) | 0.298 | 71.4 | 19.4 |  |
| Polyunsaturated Fat (g/day) | -0.337 | 13.88 | 3.76 |  |
| Saturated Fat (g/day) | 0.373 | 28.6 | 8 |  |
| Monounsaturated Fat (g/day) | -0.009 | 27 | 6.1 |  |
| Trans Fatty Acid (g/day) | 0.229 | 3.15 | 3.75 |  |
| ω-3 Fatty Acids (g/day) | -0.436 | 1.06 | 1.06 |  |
| ω-6 Fatty Acids (g/day) | -0.159 | 10.8 | 7.5 |  |
| Alcohol (g/day) | -0.278 | 13.98 | 3.72 | Dietary Components |
| Dietary Fiber (g/day) | -0.663 | 18.8 | 4.9 |  |
| Cholesterol (mg/day) | 0.11 | 279.4 | 51.2 |  |
| Tea (g/day) | -0.536 | 1.69 | 1.53 |  |
| β-Carotene (μg/day) | -0.584 | 3718 | 1720 | Vitamins |
| Vitamin B12 (μg/day) | 0.106 | 5.15 | 2.7 |  |
| Vitamin B6 (mg/day) | -0.365 | 1.47 | 0.74 |  |
| Niacin (mg/day) | -0.246 | 25.9 | 11.77 |  |
| Thiamin (mg/day) | -0.098 | 1.7 | 0.66 |  |
| Riboflavin (mg/day) | -0.068 | 1.7 | 0.79 |  |
| Vitamin A (RE/day) | -0.401 | 983.9 | 518.6 |  |
| Vitamin C (mg/day) | -0.424 | 118.2 | 43.46 |  |
| Vitamin D (mcg/day) | -0.446 | 6.26 | 2.21 |  |
| Vitamin E (mg/day) | -0.419 | 8.73 | 1.49 |  |
| Folate (μg/day) | -0.19 | 273 | 70.7 |  |
| Selenium (μg/day) | -0.191 | 67 | 25.1 | Minerals |
| Iron (Fe, mg/day) | 0.032 | 13.35 | 3.71 |  |
| Magnesium (Mg, mg/day) | -0.484 | 310.1 | 139.4 |  |
| Zinc (Zn, mg/day) | -0.313 | 9.84 | 2.19 |  |

*Positive scores indicate a pro-inflammatory effect, while negative scores indicate an anti-inflammatory effect for the corresponding food parameters; SD, standard deviation.

**Table S2. ICD-10 Codes for Participant Identification**

This table lists the ICD-10 codes employed to exclude participants with pre-existing chronic liver disease (CLD) at baseline, as well as to identify participants with CLD based on diagnoses documented in hospital and cancer records.

| **ICD-10** | **Description** |
| --- | --- |
| B18.0, B18.1, B18.2, B18.8, B18.9 | Chronic viral hepatitis B with delta-agent, Chronic viral hepatitis B without delta-agent, Chronic viral hepatitis C, Other chronic viral hepatitis, Chronic viral hepatitis, unspecified |
| B19.0, B19.9 | Unspecified viral hepatitis with coma, Unspecified viral hepatitis without coma |
| B58.1 | Toxoplasma hepatitis |
| B25.1 | Cytomegaloviral hepatitis |
| C22.0, C22.1, C22.2, C22.3, C22.4, C22.7, C22.9 | Liver cell carcinoma, Intrahepatic bile duct carcinoma, Hepatoblastoma, Angiosarcoma of liver, Other sarcomas of liver, Other specified carcinomas of liver, Liver, unspecified (malignancy) |
| C78.7 | Secondary malignant neoplasm of liver |
| D01.5, D37.6 | Liver, gallbladder and bile ducts (carcinoma), Liver, gallbladder and bile ducts (neoplasm of uncertain significance) |
| I85.0, I85.9, I86.4 | Oesophageal varices with bleeding, Oesophageal varices without bleeding, Gastric varices |
| I98.2, I98.3 | Oesophageal varices in diseases classified elsewhere, Oesophageal varices with bleeding in diseases classified elsewhere |
| K70.0, K70.1, K70.2, K70.3, K70.4, K70.9 | Alcoholic fatty liver, Alcoholic hepatitis, Alcoholic fibrosis and sclerosis of liver, Alcoholic cirrhosis of liver, Alcoholic hepatic failure, Alcoholic liver disease, unspecified |
| K71.0, K71.1, K71.2, K71.3, K71.4, K71.5, K71.6, K71.7, K71.8, K71.9 | Toxic liver disease with cholestasis, Toxic liver disease with hepatic necrosis, Toxic liver disease with acute hepatitis, Toxic liver disease with chronic persistent hepatitis, Toxic liver disease with chronic lobular hepatitis, Toxic liver disease with chronic active hepatitis, Toxic liver disease with hepatitis, not elsewhere classified, Toxic liver disease with fibrosis and cirrhosis of liver, Toxic liver disease with other disorders of liver, Toxic liver disease, unspecified |
| K72.0, K72.1, K72.9 | Acute and subacute hepatic failure, Chronic hepatic failure, Hepatic failure, unspecified |
| K73.0, K73.1, K73.2, K73.8, K73.9 | Chronic persistent hepatitis, not elsewhere classified, Chronic lobular hepatitis, not elsewhere classified, Chronic active hepatitis, not elsewhere classified, Other chronic hepatitis, not elsewhere classified, Chronic hepatitis, unspecified |
| K74.0, K74.1, K74.2, K74.3, K74.4, K74.5, K74.6 | Hepatic fibrosis, Hepatic sclerosis, Hepatic fibrosis with hepatic sclerosis, Primary biliary cirrhosis, Secondary biliary cirrhosis, Biliary cirrhosis, unspecified, Other and unspecified cirrhosis of liver |
| K75.0, K75.1, K75.2, K75.3, K75.4, K75.8, K75.9 | Abscess of liver, Phlebitis of portal vein, Nonspecific reactive hepatitis, Granulomatous hepatitis, not elsewhere classified, Autoimmune hepatitis, Other specified inflammatory liver diseases, Inflammatory liver disease, unspecified |
| K76.0, K76.1, K76.2, K76.3, K76.4, K76.5, K76.6, K76.7, K76.8, K76.9 | Fatty (change of) liver, not elsewhere classified, Chronic passive congestion of liver, Central haemorrhagic necrosis of liver, Infarction of liver, Peliosis hepatis, Hepatic veno-occlusive disease, Portal hypertension, Hepatorenal syndrome, Other specified diseases of liver, Liver disease, unspecified |
| K77.0, K77.8 | Liver disorders in infectious and parasitic diseases classified elsewhere, Liver disorders in other diseases classified elsewhere |
| O26.6 | Liver disorders in pregnancy, childbirth and the puerperium |
| Q44.6, Q44.7 | Cystic disease of liver, Other congenital malformations of liver |
| T86.4 | Liver transplant failure and rejection |
| Z52.6, Z94.4 | Liver donor, Liver transplant status |
| P35.3 | Congenital viral hepatitis |
| Z22.5 | Carrier of viral hepatitis |
| ICD-10, International Classification of Diseases 10th Revision | |

**Table S3. HEI-2020 Food Components and Scoring Criteria**

The Healthy Eating Index 2020 (HEI-2020) includes 13 sections with a maximum score of 100 points. It is divided into adequacy components, which highlight foods to consume more of for better health, and moderation components, which focus on foods to limit for good health. Scores for intakes between minimum and maximum standards are assigned proportionately. The final HEI-2020 score is the sum of all component scores, reflecting overall dietary quality.

| **Component**  **Adequacy** | **Maximum Points** | **Standard for Maximum Score** | **Standard for Minimum Score of Zero** |
| --- | --- | --- | --- |
| Total Fruits^1^ | 5 | ≥0.8 cup equiv. per 1,000 kcal | No Fruits |
| Whole Fruits^2^ | 5 | ≥0.4 cup equiv. per 1,000 kcal | No Whole Fruits |
| Total Vegetables^3^ | 5 | ≥1.1 cup equiv. per 1,000 kcal | No Vegetables |
| Greens and Beans^3^ | 5 | ≥0.2 cup equiv. per 1,000 kcal | No Dark Green Vegetables or Legumes |
| Whole Grains | 10 | ≥1.5 oz equiv. per 1,000 kcal | No Whole Grains |
| Dairy^4^ | 10 | ≥1.3 cup equiv. per 1,000 kcal | No Dairy |
| Total Protein Foods^3^ | 5 | ≥2.5 oz equiv. per 1,000 kcal | No Protein Foods |
| Seafood and Plant Proteins^5^ | 5 | ≥0.8 oz equiv. per 1,000 kcal | No Seafood or Plant Proteins |
| Fatty Acids^6^ | 10 | (PUFAs + MUFAs)/SFAs ≥2.5 | (PUFAs + MUFAs)/SFAs ≤1.2 |
| **Moderation:** |  |  |  |
| Refined Grains | 10 | ≤1.8 oz equiv. per 1,000 kcal | ≥4.3 oz equiv. per 1,000 kcal |
| Sodium | 10 | ≤1.1 gram per 1,000 kcal | ≥2.0 grams per 1,000 kcal |
| Added Sugars | 10 | ≤6.5% of energy | ≥26% of energy |
| Saturated Fats | 10 | ≤8% of energy | ≥16% of energy |
| **Score** | 0-100 points total (13 components: 0–5 points or 0-10 points each) | | |

1 Includes 100% fruit juice.

2 Includes all forms except juice.

3 Includes legumes (beans and peas).

4 Includes all milk products, such as fluid milk, yogurt, and cheese, and fortified soy beverages.

5 Includes seafood, nuts, seeds, soy products (other than beverages), and beans, peas, and lentils.

6 Ratio of poly- and monounsaturated fatty acids (PUFAs and MUFAs) to saturated fatty acids (SFAs).

**Table S4. Mediterranean Diet Score Components and Scoring Standards**

The Mediterranean diet score comprises nine components. One point is awarded for intakes at or above the cohort-specific median for vegetables, fruits, whole grains, nuts, legumes, fish, and the ratio of monounsaturated to saturated fat. An additional point is given for intakes below the cohort-specific median for red and processed meats, and for alcohol consumption ranging from 5-15 g/d for women and 10-25 g/d for men. The total score ranges from 0 to 9 points, reflecting adherence to Mediterranean dietary pattern.

| **Component** | **1 point for individuals who meet each standard** |
| --- | --- |
| Vegetables | Above or equal to the median |
| Fruits | Above or equal to the median |
| Whole Grains | Above or equal to the median |
| Nuts | Above or equal to the median |
| Legumes | Above or equal to the median |
| Fish | Above or equal to the median |
| Ratio of Monounsaturated to Saturated Fat | Above or equal to the median |
| Red and Processed Meat | Below median |
| Alcohol Intake | 5-15 g/d for women and 10-25 g/d for men |
| **Score** | 0-9 points total |

**Table S5. Associations of eDII and DII from Typical Dietary Participants with CLD Risk**

This table presents the association between the eDII and the DII from participants with typical dietary patterns, and their respective risks of developing CLD. The analysis is divided into quartiles (Q1-Q4) and assessed continuously, with trends tested for significance. Hazard ratios, with 95% confidence intervals and corresponding P-values, are shown for three models: Model 1 adjusts for age, sex, ethnicity, education, and the Townsend deprivation index. Model 2 includes these variables plus drinking status, smoking status, body mass index, and physical activity. Model 3 further adjusts for diabetes and blood pressure status.

|  | **Q1** | **Q2** | **Q3** | **Q4** | **Continous** | **P for trend** |
| --- | --- | --- | --- | --- | --- | --- |
| **eDII (Cases/Participants)** | 945/30331 | 919/30331 | 981/ 30331 | 1173/30331 |  |  |
| Model1 | 1 (reference) | 0.974 (0.889-1.067) 0.566 | 1.034 (0.945-1.132) 0.467 | 1.237 (1.133-1.351) < 0.001 | 1.056 (1.037-1.075) < 0.001 | < 0.001 |
| Model2 | 1 (reference) | 0.958 (0.874-1.049) 0.354 | 0.99 (0.904-1.084) 0.827 | 1.124 (1.029-1.228) 0.009 | 1.033 (1.014-1.052) < 0.001 | 0.007 |
| Model3 | 1 (reference) | 0.962 (0.878-1.053) 0.4 | 0.996 (0.91-1.091) 0.939 | 1.138 (1.042-1.244) 0.004 | 1.036 (1.017-1.054) < 0.001 | 0.003 |
| **Typical Dietary (Cases/Participants)** | 834/26058 | 771/24921 | 804/24309 | 898/23399 |  |  |
| Model1 | 1 (reference) | 0.989 (0.896-1.090) 0.818 | 1.065 (0.967-1.174) 0.204 | 1.248 (1.135-1.373) < 0.001 | 1.048 (1.030-1.067) < 0.001 | < 0.001 |
| Model2 | 1 (reference) | 0.992 (0.898-1.096) 0.869 | 1.031 (0.934-1.138) 0.545 | 1.149 (1.044-1.266) 0.005 | 1.030 (1.012-1.048) 0.001 | 0.003 |
| Model3 | 1 (reference) | 0.992 (0.898-1.096) 0.880 | 1.032 (0.936-1.139) 0.525 | 1.152 (1.047-1.269) 0.004 | 1.030 (1.012-1.049) 0.001 | 0.003 |
| DII, dietary inflammatort index; eDII; energy-adjuested DII | | | | | | |

**Table S6. Relationship between HEI-2020 and CLD Risk**

This table presents the association between the HEI-2020 and the risk of CLD across quartiles (Q1-Q4) and as a continuous variable, with trends tested for significance. The analysis includes three models: Model 1 adjusts for age, sex, ethnicity, education, and the Townsend deprivation index. Model 2 includes additional adjustments for drinking status, smoking status, body mass index, and physical activity. Model 3 further considers diabetes and blood pressure status. Hazard ratios (HRs) are reported from the Cox regression models, while the accelerated failure time models provide adjusted time ratios derived from model coefficients. The data presented include HRs, 95% confidence intervals, and P-values.

| **HEI-2020** | **Q1** | **Q2** | **Q3** | **Q4** | **Continous** | **P for trend** |
| --- | --- | --- | --- | --- | --- | --- |
| **CLD** |  |  |  |  |  |  |
| Model1 | 1 (reference) | 0.954 (0.876-1.039) 0.277 | 0.877 (0.803-0.957) 0.003 | 0.838 (0.767-0.916) < 0.001 | 0.992 (0.989-0.995) < 0.001 | < 0.001 |
| Model2 | 1 (reference) | 0.985 (0.904-1.072) 0.722 | 0.923 (0.846-1.007) 0.0726 | 0.907 (0.829-0.992) 0.032 | 0.995 (0.992-0.999) 0.004 | 0.015 |
| Model3 | 1 (reference) | *0.987 (0.927-1.054) 0.647 | *0.937 (0.880-1.000) 0.049 | *0.923 (0.860-0.992) 0.016 | 0.995 (0.992-0.998) 0.002 | 0.007 |
| *Accelerated Failure Time model | | | | | | |

**Table S7. Relationship between MEDS and CLD Risk**

This table presents the hazard ratios with 95% confidence intervals and P-values for the association between the MEDS and disease risk, analyzed using a Cox regression model. Model 1 adjusts for age, sex, ethnicity, education, and the Townsend deprivation index. Model 2 includes additional adjustments for drinking status, smoking status, body mass index, and physical activity. Model 3 further considers diabetes and blood pressure status.

| **MEDS** | **HR 95%CI** | **P** |
| --- | --- | --- |
| Model1 | 0.941 (0.921-0.961) | < 0.001 |
| Model2 | 0.961 (0.941-0.982) | < 0.001 |
| Model3 | 0.961 (0.941-0.982) | < 0.001 |

#### 2 Supplementary Figures

**Figure S1. Nonlinear Associations Between Dietary Patterns and Risk of CLD and Cirrhosis**

This figure illustrates the association between dietary patterns, specifically the HEI-2020 and MEDS, and the risk of CLD and cirrhosis. Panels A and B show the HEI-2020's effect on hazard ratios (HRs), with A representing CLD and B representing cirrhosis. Panels C and D display the impact of the MEDS, with C representing CLD and D representing cirrhosis. The blue lines indicate the estimated HRs, while the shaded areas denote the 95% confidence intervals. Both overall and nonlinear P-values assess the significance of the associations and any deviations from linearity.


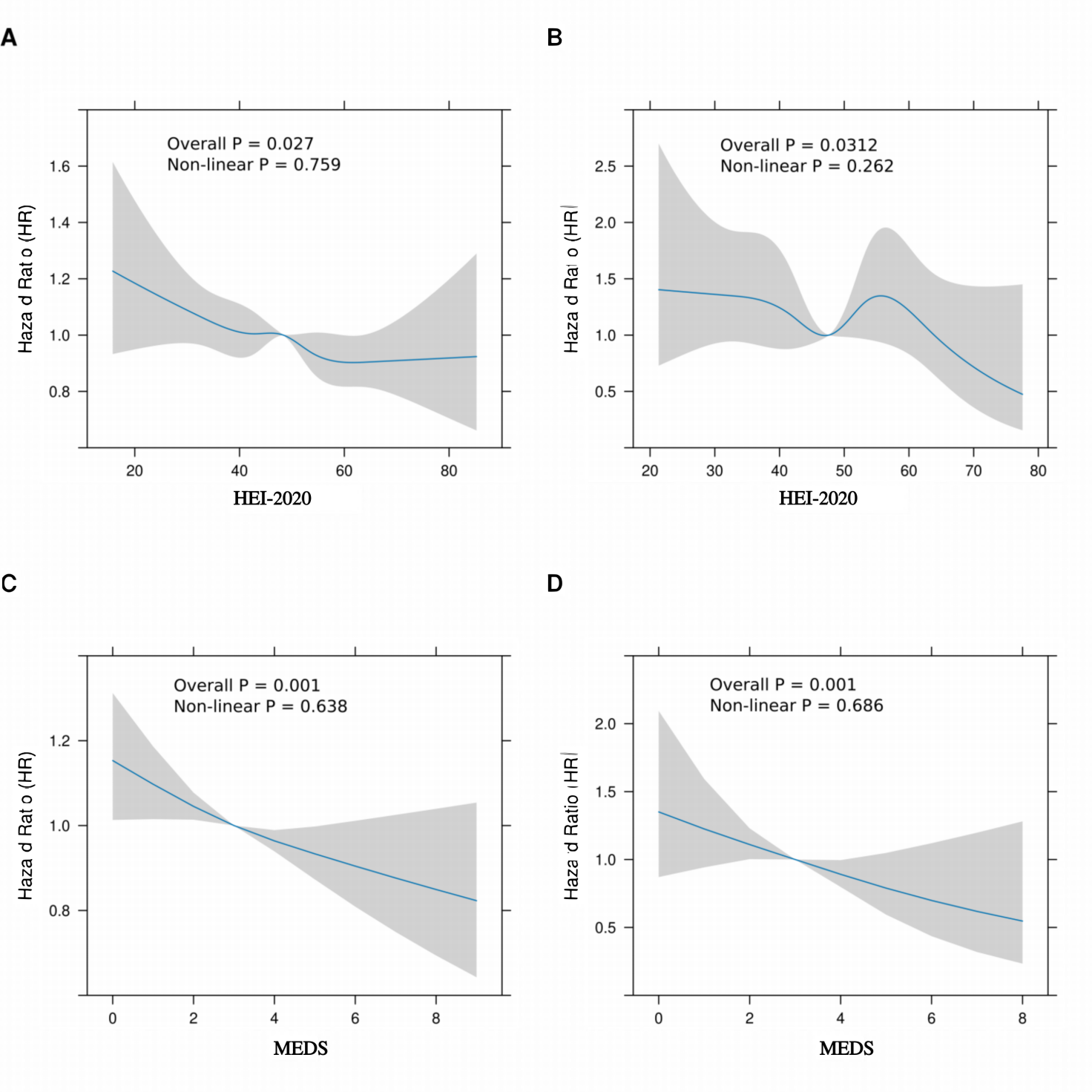

Supplement: Supplementary file 1 [file Data_Sheet_1.docx]
